# Expressional and Prognostic Value of S100A16 in Pancreatic Cancer *Via* Integrated Bioinformatics Analyses

**DOI:** 10.3389/fcell.2021.645641

**Published:** 2021-04-12

**Authors:** Gangping Tu, Wenzhe Gao, Ying Li, Yating Dian, Bingyang Xue, Li Niu, Xiao Yu, Hongwei Zhu

**Affiliations:** ^1^Department of Hepatopancreatobiliary Surgery, The Third Xiangya Hospital, Central South University, Changsha, China; ^2^Medical College of Xiangya, Central South University, Changsha, China

**Keywords:** S100A16, pancreatic adenocarcinoma, TCGA, prognosis, immune infiltration

## Abstract

Studies have shown that the calcium-binding protein family S100 may play a role in the development of pancreatic cancer (PC), but the role of S100A16 in PC is still unknown. In this study, Oncomine was first used to detect the expression level and prognosis of S100A16 in PC and other tumors. The results showed that S100A16 was highly expressed in PC tissues compared with a normal pancreas, and the increased expression level may be related to poor prognosis in PC patients. The TCGA and ICGC RNA-seq data of PC patients were downloaded, and the S100A16-related differentially expressed genome (DEGs) was defined by taking the intersection of two gene sets. The GO and KEGG pathways were then analyzed. For clinical analysis, boxplots were depicted for the correlation between clinical characteristics and S100A16 expression. Then Cox regression was applied for exploring the prognostic value of S100A16 for PDAC patients. Based on the Cox regression model, we further estabished a S100A16-related risk score system to strengthen the ability to predict patients' prognosis. After integrating the risk score model and multiple clinicopathological factors, we finally established a nomogram that could predict the survival time of patients. Moreover, Gene set enrichment the effect of S100A16 expression differences on downstream biological processes. At last, using TIMER, ImmuneCellAI and GSEA we analyzed the correlation between S100A16 and pancreatic cancer immune infiltration and predicted the response of patients to checkpoint Blocker (ICB). In summary, S100A16 is involved in the occurrence and development of PC, affecting the prognosis of patients, and may have potential reference values for the immunotherapy of PC.

## Introduction

Pancreatic cancer (PC) is a kind of disease with an extremely high degree of malignancy (Tempero, 2019). Although progress in clinical drug therapy has been made, due to the interaction between tumor cells and microenvironment, it is still highly resistant to radiotherapy and chemotherapy. Moreover, PC is often difficult to diagnose at the early stages, but it is prone to exert early metastasis. Generally speaking, the prognosis is still poor for PC patients (Vincent et al., 2011). Further research on the driving genes of PC and the proteins in the tumor microenvironment that enhance the interaction between invasion and metastasis will help guide the development of new treatment methods, decipher treatment resistance, and predict and monitor treatment response (Ligorio et al., 2019).

S100 proteins are a calcium-binding protein. Its molecular structure consists of antiparallel homopolymers and heterodimers. Each monomer is composed of two helical loops connected by a hinge region (EF-1 and EF-2). S100 proteins are widely expressed in different organs and tissues. At present, there are 25 known members in the S100 protein family (Donato, 2001). These proteins play an important role in the basic processes of cell proliferation, apoptosis, differentiation, and inflammation through participating in a variety of different pathways (Donato et al., 2017). Furthermore, its role in tumors is also worth further attention. The function of S100 proteins in breast cancer, lung cancer, and malignant melanoma has been studied to a certain extent. The pathological signal of S100 can also be observed in PC (Bresnick et al., 2015). S100A2, S100A4, S100A8, S100A9, and S100A11 in the S100 family have been found to be associated with the pathogenesis and invasion of PC (Allgöwer et al., 2020). However, the effect of the S100 protein family in PC needs to be further explored.

**Figure 1:**
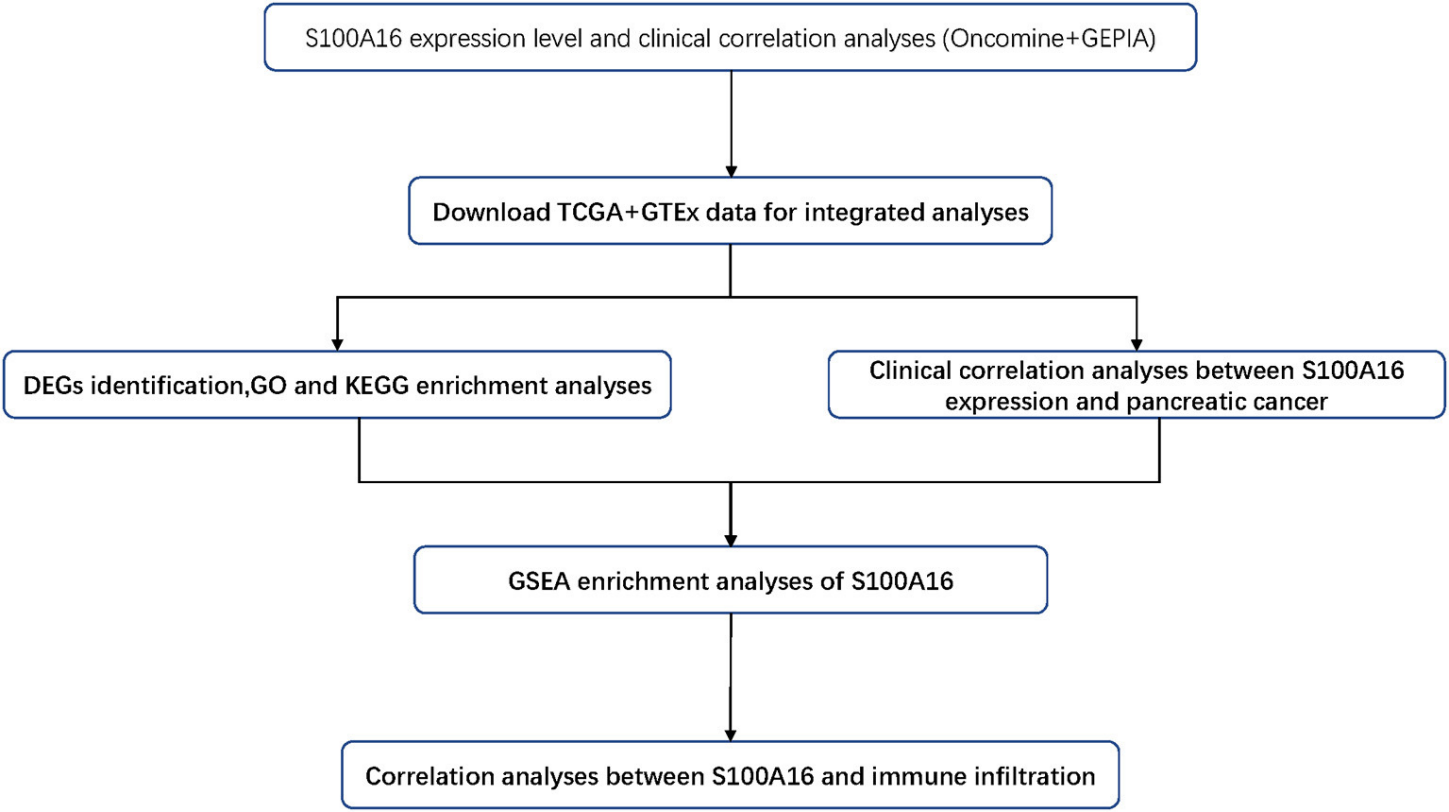
The pipeline designed for this work. TCGA, The Cancer Genome Atlas, DEGs: differentially expressed gene; GO, gene ontology; KEGG, Kyoto Encyclopedia of Genes and Genomes; GSEA, gene set enrichment analysis.

It has been reported that S100A16 is linked to obesity, type 2 diabetes, and inflammation through the calcium dependent mechanism (Gonzalez et al., 2020). In addition, it has also been found to be linked to a number of tumors, including carcinoma of the urinary bladder, lung cancer, thyroid gland cancer, ovarian cancer, and gastric cancer (Zhu et al., 2016; Chen et al., 2018; Sun et al., 2018). S100A16 participates in various signal adjustment pathways, such as extracellular signal-regulated kinase, Notch, and nuclear factor kappa B pathways. Research by Zhu et al. (2016) indicate that the overexpression of S100A16 adjusts protein kinase transcription factors according to Akt and extracellular data signals to promote the proliferation and erosion of cancer cells. Despite a certain degree of understanding, the exact biological function of S100A16 in PC is still unclear. This article explores the role of S100A16 in PC through a bioinformatics analysis. The workflow chart is shown in Figure 1.

## Results

### The Expression of S100A16 Increased in PDAC Tissues

**Figure 2:**
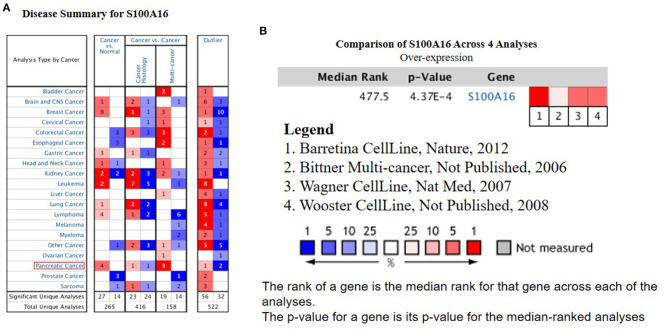
High expression of S100A16 in PC based on Oncomine**. (A)** In PC, S100A16 was significantly upregulated in four studies. Blue represents low expression. Red represents high expression. The darker the color, the greater the significance. **(B)** Comparison of S100A16 expression across four analyses, and red means high expression. *P* < 0.01.The tumor type represented by each number is shown in Supplementary Figure 1.

**Figure 3:**
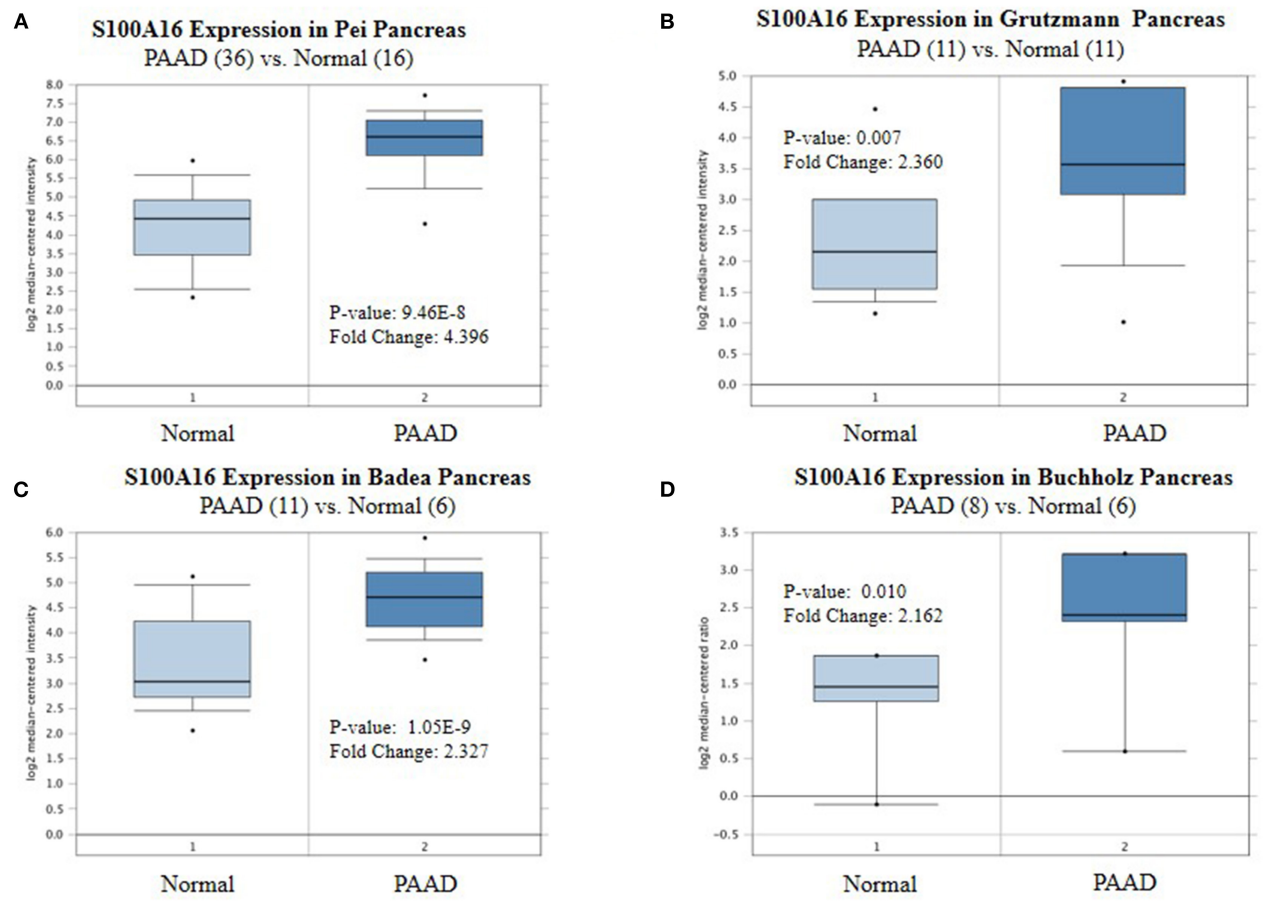
Expression level of S100A16 in each study based on Oncomine. **(A)** Pei pancreas, **(B)** Grutzmann Pancreas, **(C)** Badea Pancreas, **(D)** Buchholz Pancreas, Top 10% refers to the top10% of genes; *P* < 0.01.

**Figure 4:**
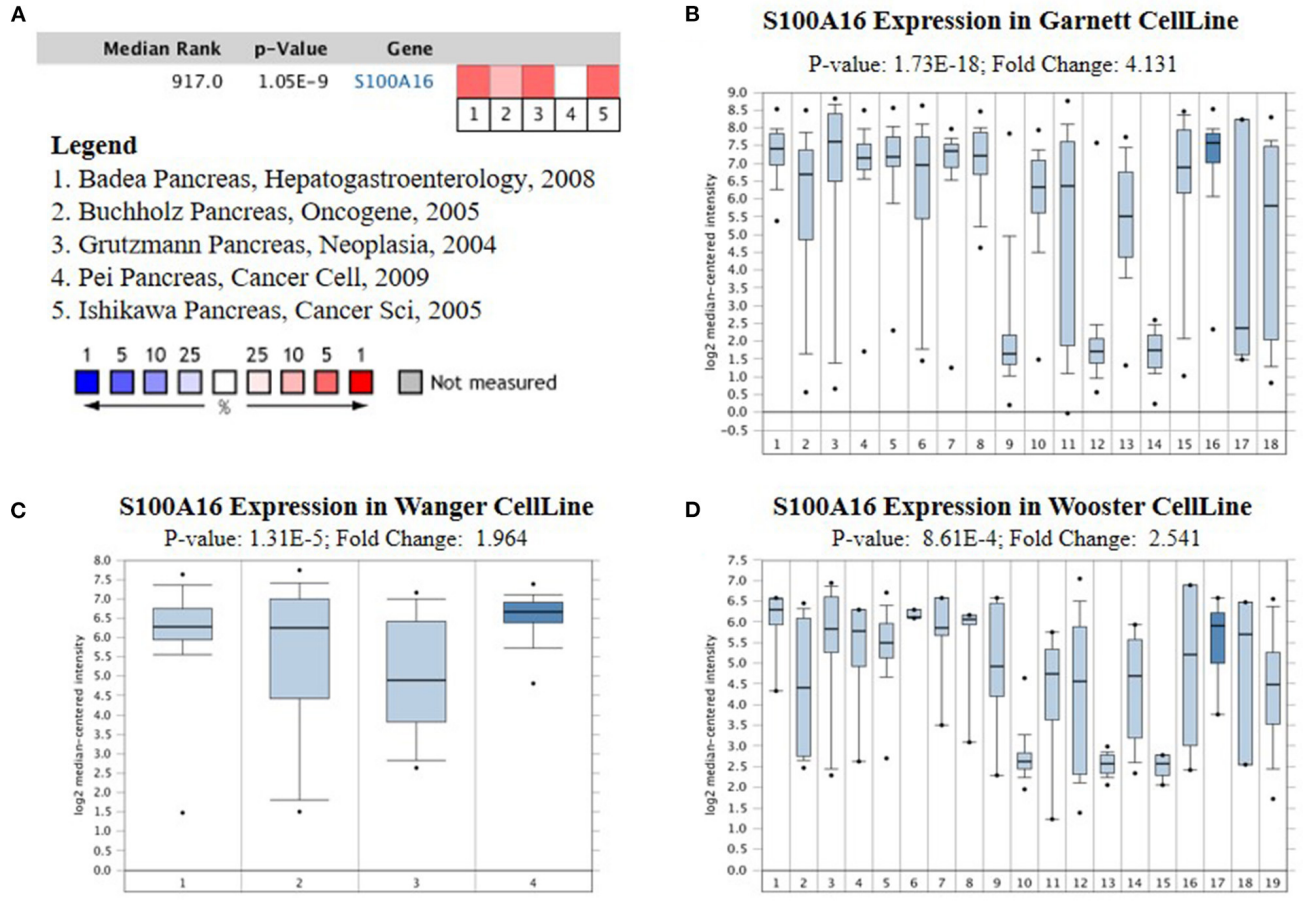
Analysis of relationship between the S100A16 expression and the type of PC. **(A)** Comparison of S100A16 expression across five analyses, and red means high expression. S100A16 expression in **(B)** Garnett cell Line**, (C)** Wanger cell line **(D)** Wooster cell line. The expression level is evaluated *via* the median line; Top 10% refers to the top 10% of genes; *P* < 0.01.

**Figure 5:**
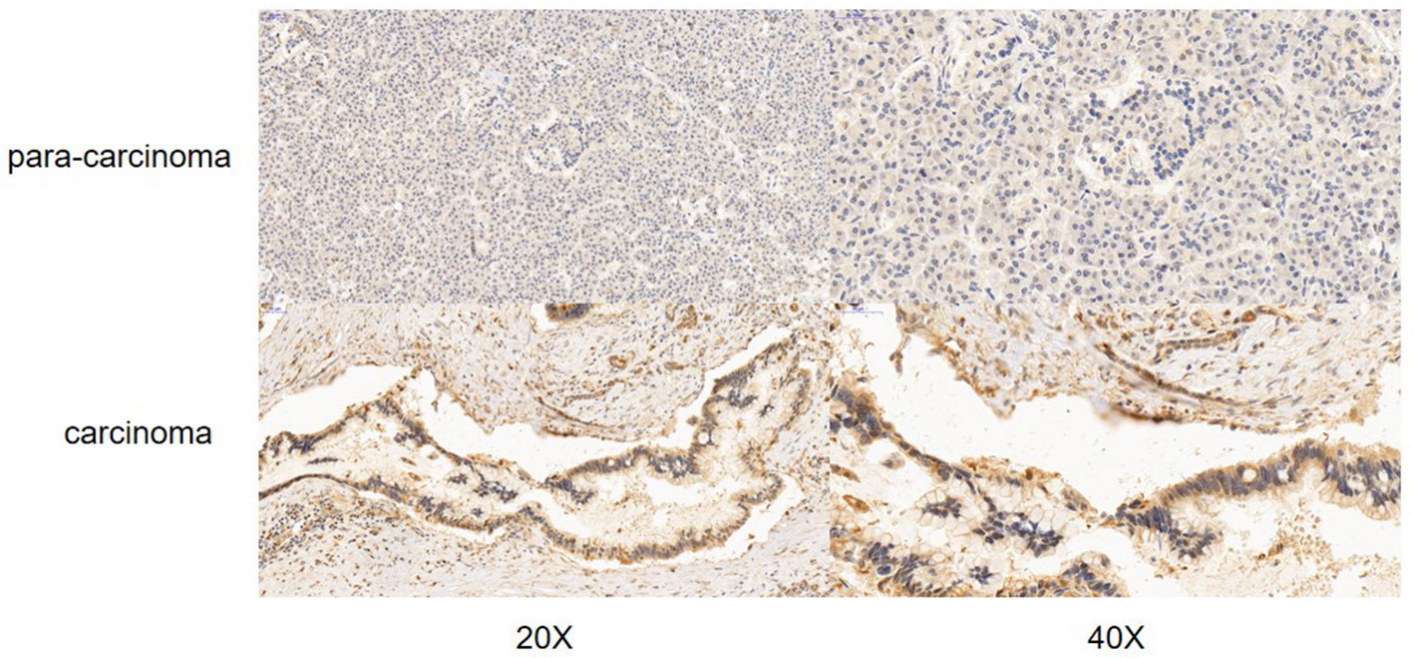
Immunohistochemistry of S100A16 in PC tissues and adjacent tissues.

According to the Oncomine database query, the analysis of cancer and all normal comparisons, contains 265 different scientific studies on S100A16. In these studies, four studies on the up-regulation of S100A16 in PC were found, but no down-regulation study was found, suggesting that S100A16 may be up-regulated in PC. In addition, among the 158 multi-cancer comparison studies, there were three studies in pancreatic cancer that were significantly up-regulated. Apart from this, there were 522 outlier studies, including one study on the up regulation of S100A16 and two studies on the down regulation of S100A16 (Figure 2A). Four studies involving the differential expressions of S100A16 in the Oncomine database between Pancreatic ductal adenocarcinoma (PDAC) and normal tissues were screened (Figures 3A–D). A comprehensive analysis of the four scientific studies that met the selection criteria showed that the negative correlation level was 477.5, and the *p*-value was 4.37e−4, indicating that compared with all normal tissues, the expression level of S100A16 in pancreatic tumors increased (Figure 2B, *P* < 0.01). In addition, in every scientific study, it has also been found that S100A16 in pancreatic tumors is higher than that in all normal tissues. To further clarify whether the expression of S100A16 is different from that in other malignant tumors, an analysis of cancer vs. cancer studies was carried out. There are five scientific studies that meet the selection criteria, with a negative correlation ranking of 917.0 and a *p*-value of 1.05e−9 (Figure 4A). The data shows that S100A16 in pancreatic tumors is significantly higher than other types of malignant tumors (Figures 4B–D). This result shows that S100A16 is highly expressed in PDAC patients. It is likely to play a key buffering role for S100A16 in the carcinogenesis and development of pancreatic tumors. Pancreatic carcinoma and adjacent normal pancreatic tissues from three patients were collected and immunohistochemically assayed. Figure 5 shows the S100A16 immunohistochemical results of one of the patients and the S100A16 immunohistochemical results of another two patients have been added to the Supplementary Material (Figure 5 and Supplementary Figure 2). These results confirmed the above conclusion that S100A16 was highly expressed in pancreatic cancer tissues through a database analysis.

### Relationship Between S100A16 Expression and Prognosis of Patients With PDAC

**Figure 6:**
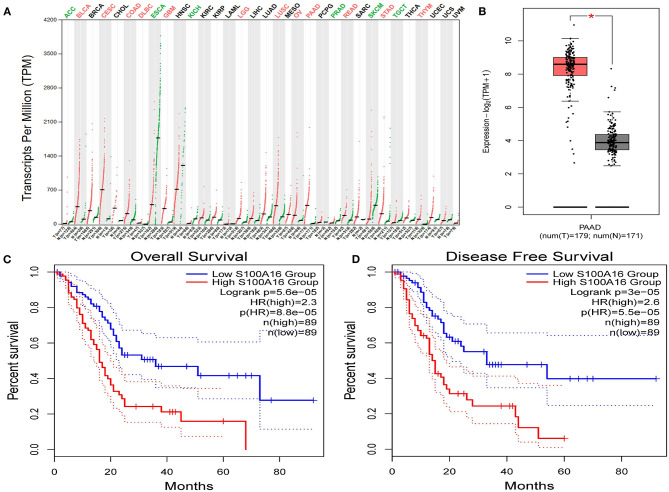
Relationship between S100A16 expression and prognosis of PC patients based on GEPIA database. **(A)** S100A16 was significantly upregulated in various tumors, such as BLCA, CESC, COAD.DLBC, LGG,OV, PAAD, READ, and THYM. Expression level of S100A16 in PAAD in comparison with the normal control. *P < 0.05. **(B)** The relationship between S100A16 expression levels and overall, in PDAC patients as analyzed by GEPIA database. **(C)** The relationship between S100A16 expression levels and disease-free survival in PC patients as analyzed by GEPIA database and disease-free survival based on GEPIA database **(D)**.

Screening GEPIA database information showed that S100A16 expression was up-regulated in BLCA (Bladder Urothelial Carcinoma), CESC (Cervical squamous cell carcinoma and endocervical adenocarcinoma), COAD (Colon adenocarcinoma), DLBC (Lymphoma Diffuse Large B Somatic Lymphoma), GBM (Glioblastoma multiforme), LGG (Brain Lower Grade Glioma), LUSC (Lung squamous cell carcinoma), OV (Ovarian serous cystadenocarcinoma), PAAD (pancreatic tumor), READ (Rectum adenocarcinoma), STAD (rectal cancer), THYM (thymoma). The expression of S100A16 was down-regulated in ACC (Adrenocortical carcinoma), ESCA (Esophageal carcinoma), KICH (Kidney Chromophobe), PRAD (Prostate adenocarcinoma), SKCM (Skin Cutaneous Melanoma), and TGCT (Testicular Germ Cell Tumors) (Figure 6A). The association between S100A16 expression and PDAC was further elucidated, and the results were consistent with the above Oncomine analysis (Figure 6B). In addition, the prognostic analysis showed that high expression of S100A16 jeopardized the disease-free survival and overall survival of PADC patients (Figures 6C,D *P* < 0.05).

### GO Analysis and KEGG Analysis of Differentially Expressed Genes

**Figure 7:**
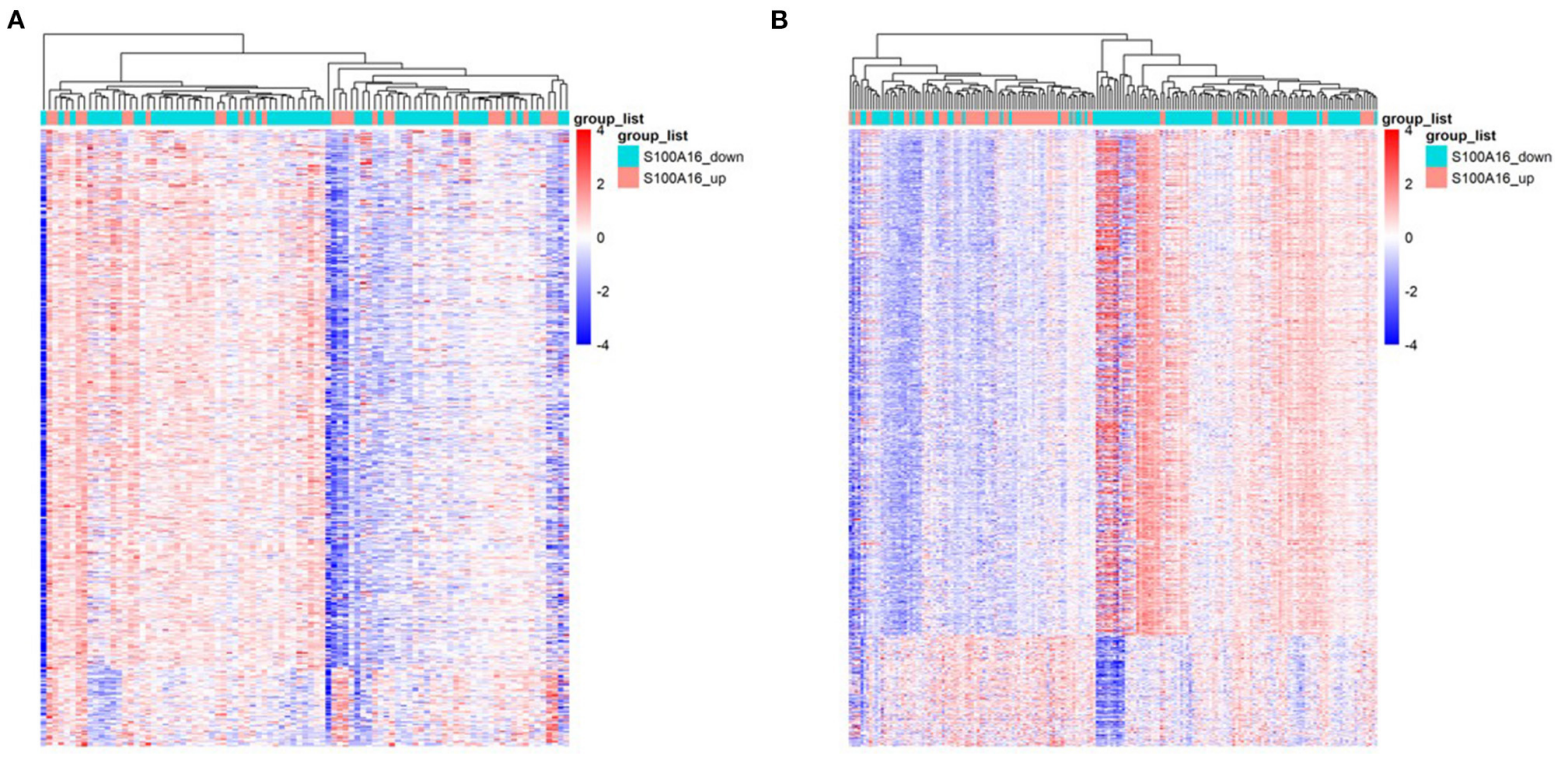
Heat map of DEGs when S100A16 is down-regulated and up-regulated in PDAC based on TCGA database **(A)** and ICGC database **(B)**.

**Figure 8:**
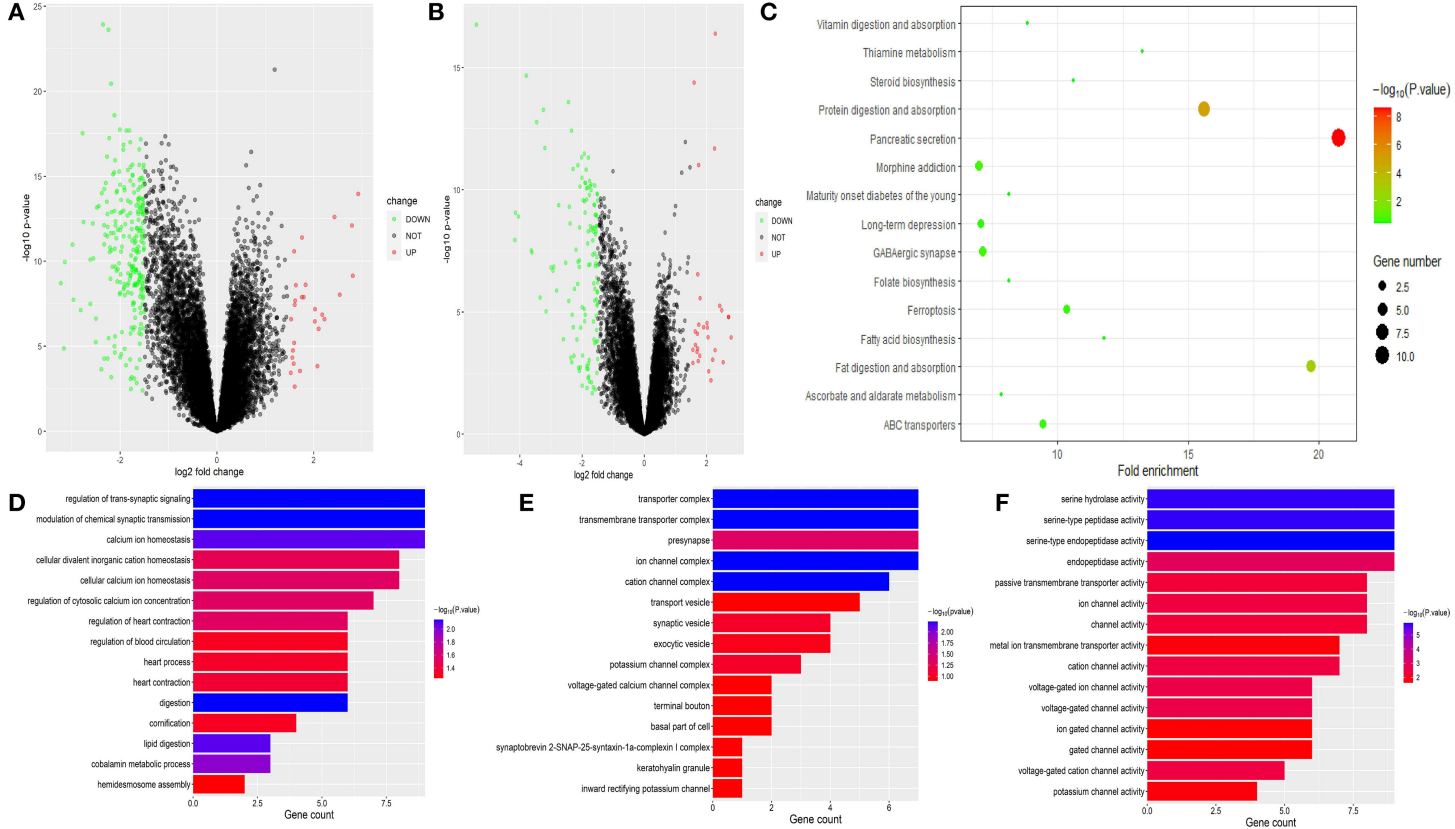
Volcanic map based on differential gene expressions of TCGA **(A)** and ICGC. **(B)** KEGG pathway analysis of DEGs. Only pathways with a *P*-value< 0.05 are presented. **(C)** GO analysis of DEGs in PC Biological process, cell components, and molecular function enrichment analyses of DEGs **(D–F)**.

Through differential expression gene analysis, a total of 1330 DEGs (|logFC|> 2, FDR < 0.05) were screened from TCGA and ICGC cohorts of PDAC patients (Figure 7). The volcano map was drawn according to the selected DEGs (Figures 8A,B. Then, 71 intersected genes were selected, and GO analysis of these genes showed that these DEGs were mainly concentrated in the regulation of trans-synaptic signals, the regulation of chemical synaptic transmission, and calcium homeostasis. The Transporter Complex, Transmembrane Transporters Complex, Presynapse, Serine hydrolase activity, Serine- type Peptidase activity, Serine-type endopeptidase activity, etc. are shown in Figures 8D–F.The analysis of the KEGG channel shows that the DEGs are mainly concentrated in ways relating to human fat digestion and absorption, protein digestion and absorption, and pancreatic secretion, with the pathway that is associated with pancreatic secretory function perhaps being the most important (Figure 8C).

### Clinical Correlation Between S100A16 Expression and Clinical Characteristics Based on TCGA Data

**Figure 9:**
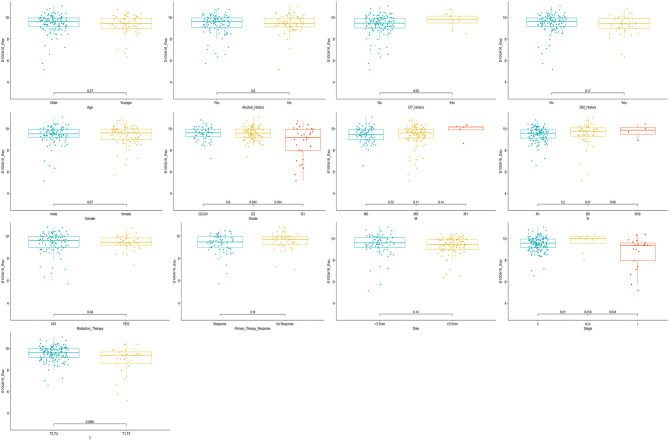
Clinical correlation analysis based on TCGA database.

**Figure 10:**
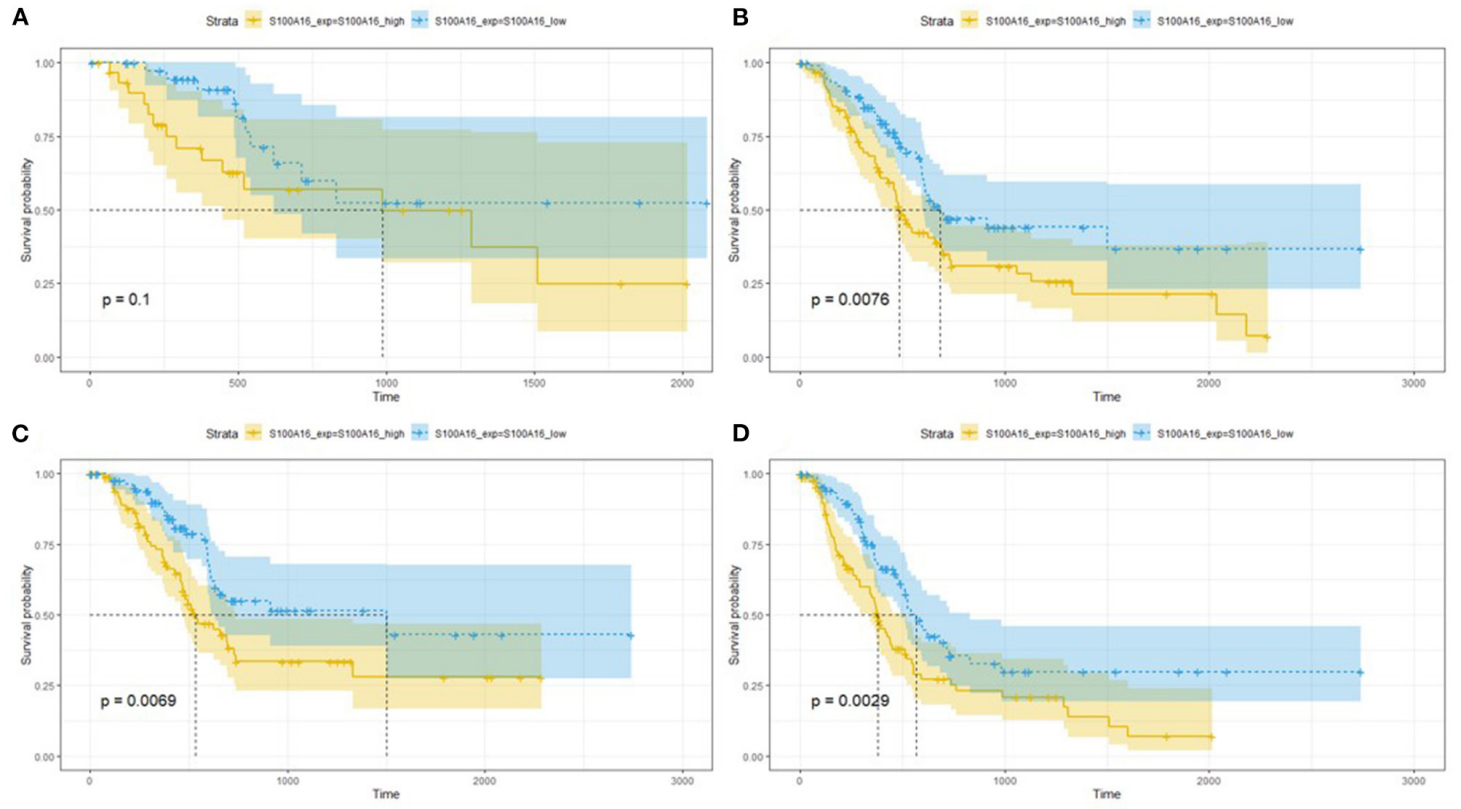
Relationship between S100A16 expression levels and survival rate in PC as analyzed by Kaplan-Meier **(A–D)**. The relationship between survival probability and S100A16 expression level.

Through the analysis of 182 TCGA specimens, it was found that high or low expression of S100A16 was significantly correlated with Primary tumor site, stage, and grade of PDAC (*P* < 0.05). It has no obvious correlation with age, size, alcohol history, gender, primary therapy Response, DM History, radiation therapy, and lymphatic metastasis (Figure 9). Kaplan-Meier analysis showed that patients in the high expression group of S100A16 had a poor prognosis (Figure 10), which is also consistent with the GEPIA analysis result. This result shows that S100A16 can be used as an index value for the progress and prognosis of pancreatic tumor patients, while the high expression of S100A16 is correlated with reduced survival time. Univariate COX analysis was adopted to verify the above conjecture, and it was found that S100A16 is a high-risk factor along with age, histological classification, primary therapy response, and Radiation therapy (HR = 1.647 95%CI = 1.24**–**2.189). However, when factors related to the survival rate of patients, analyzed by univariate COX regression analysis, were included into a multivariate COX regression analysis, S100A16 (HR = 0.632; 95% CI = 0.383 1.044) was no longer correlated with the patient's overall survival (Table 1).

**Table 1:** Univariate and multivariate analyses of prognostic factors in 182 cases of PDAC.

**Parameter**	**Univariate analysis**			**Multivariate analysis**		
	**HR**	**95%CI**	***P***	**HR**	**95%CI**	***P***
Age	1.028	1.008–1.049	0.006	1.019	1.0076–1.0483	0.007
History of CP	1.161	0.5554–2.428	0.7			
History of DM	1.119	0.5142–1.554	0.7			
Maximum tumor diameter	1.008	0.904–1.124	0.9			
Histologic grade(Grade 1,2 vs. Grades 3, 4)	1.6901	1.065–2.682	0.02	1.4087	0.9032–2.1973	0.130816
M	1.463	0.1650–2.831	0.7			
N	1.236	0.7929–1.927	0.4			
T	4.228	1.327–13.47	0.008	2.79	0.8745–8.898	0.08302
Primary therapy Response	0.444	0.2772–0.7113	3.00E-04	0.3865	0.2374–0.6292	0.000132
Radiation_Therapy	0.5354	0.292–0.981	3.00E-02	0.5383	0.2923–0.9910	0.046717
Gender_male	0.8174	0.545–1.226	0.3			
Tumor Stage III, IV	2.506	0.7917–7.924	0.1			
Alcohol exposure yes	1.186	0.7657–1.838	0.1			
S100A16 expression	1.647	1.24–2.189	6.00E−04	0.632	0.383–1.044	0.073
Risk_score	2.253	1.24–2.189	1.601–3.169	3.5053	2.0038–6.1318	1.10E−05

**Figure 11:**
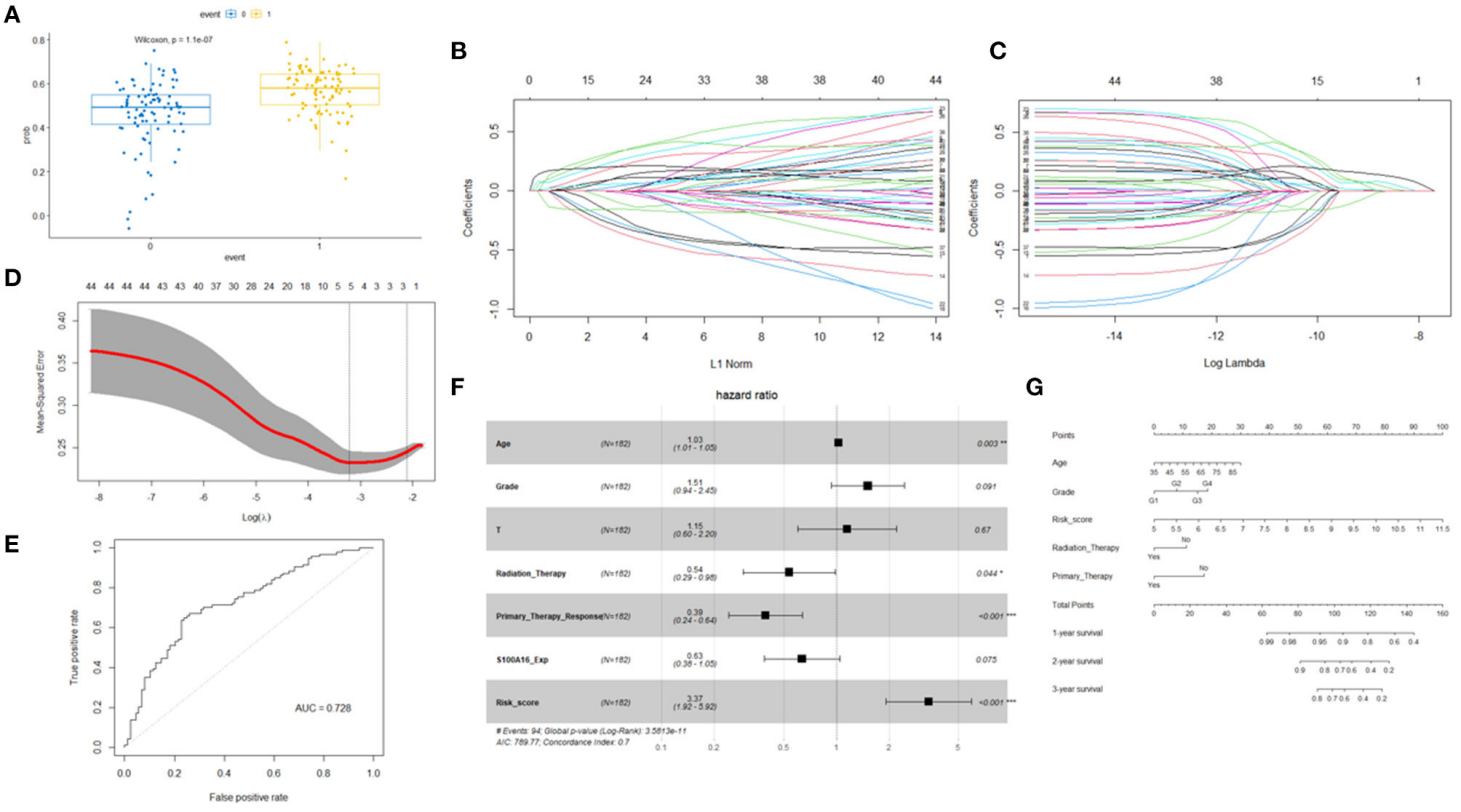
Wilcoxon signed-rank test was performed to determine whether these gene sets were related to patient prognosis. **(A)** Least absolute 1 shrinkage and selection operator (LASSO) regression was performed. **(B,C)** Calculating the minimum criteria. **(D)** ROC curves for prognostic models. **(E)** Univariate and multivariate analyses revealed that risk score was an independent prognostic predictor in the TCGA datasets. **(F)** Nomogram based on risk score, age, WHO grade, radiation therapy, and primary therapy **(G)**.

In the case where S100A16 could not predict prognosis in a multivariate cox model, we established a prognostic model by looking for prognostic molecules that are potentially related to S100A16. As mentioned in the Methods section, we first checked the expression correlation of all the genes detected in the TCGA and ICGC datasets with S100A16. Genes that possessed a Pearson correlation coefficient > 0.5 were considered as S100A16 related genes. These related genes were then included in the univariate Cox regression analysis. After these two steps, a gene set consisting of 42 S100A16 related prognostic genes was obtained. Wilcoxon signed-rank test was performed to determine whether this gene set was related to the prognosis of patients, and the results showed: Wilcoxon: *P* = 1.1E-7 (Figure 11A). To use as few genes as possible to achieve the best prognostic prediction effect, the five most representative genes, “*SERPINB1*”, “*RAB27B*”, “*MGLL*”, “*ANKRD22*”, and “*UCA1*” were obtained by applying the LASSO regression dimension reduction analysis. The risk score model was then constructed for the five molecules obtained by LASSO along with S100A16 using the algorithm mentioned in the Methods section (Figures 11B–D). The established risk score model and the aforementioned univariate cox regression factors related to prognosis were then incorporated into the multivariate Cox regression analysis again, and the results showed that the age and the risk score we established (HR = 2.253; 95%CI = 2.0038**–**6.1318) were risk factors for low survival rate of PDAC patients, while responding to primary therapy and the radiation therapy received were protective factors (Table 1). ROC curve then proved that this model had a good prediction ability, with AUC = 0.728 (Figure 11E).

To better establish a predictive tool for quantitative analysis of OS in PDAC patients in clinical work, we applied the risk score model, combined with the elements of clinical characteristics that were positive in the above multivariate cox model to build a nomogram model (Figures 11F,G). This model further illustrated the potential guiding role of the S100A16-related gene risk score in PC patients.

### GSEA Enrichment Analysis of S100A16

**Figure 12:**
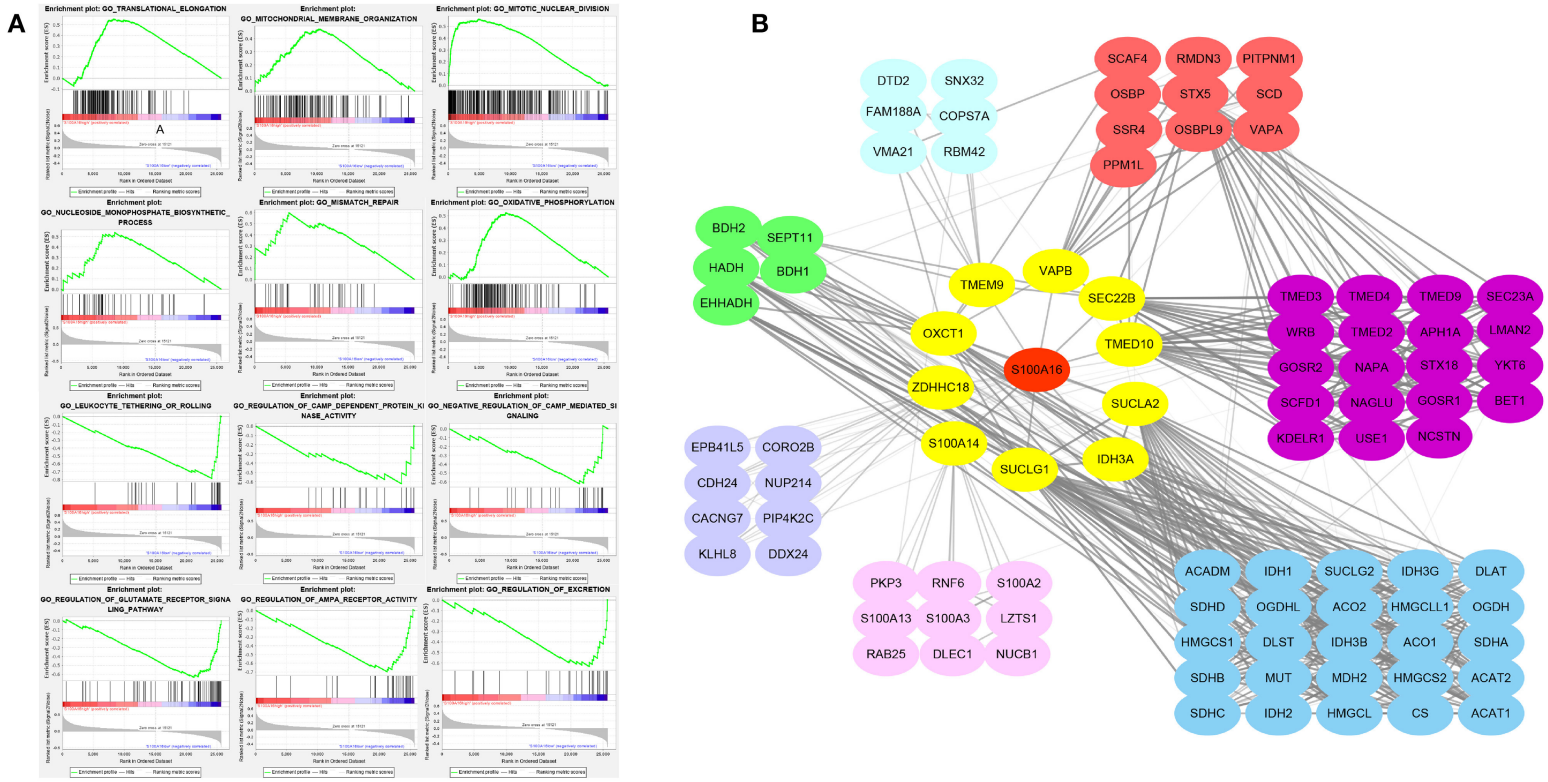
Enrichment plots from GSEA analysis. GSEA analysis showed that translational elongation, mitochondrial membrane organization, mitotic nuclear division, nucleoside monophosphate biosynthetic process, and mismatch repair oxidative phosphorylation was differentially enriched in the S100A16 high expression phenotype. Leukocyte tethering or rolling, regulation of CAMP-dependent protein kinase activity, negative regulation of CAMP-mediated signal, regulation of glutamate receptor signaling pathway, regulation of AMPA receptor activity, and regulation of excretion was differentially enriched in the low expression phenotype of S100A16. Translational elongation, mitochondrial membrane organization, mitotic nuclear division, nucleoside monophosphate biosynthetic process, and mismatch repair oxidative phosphorylation was differentially enriched in the low expression phenotype of S100A16. **(A)** Protein-protein interaction network for S100A16 in PDAC **(B)**.

To further analyze the role of S100A16 in the pancreatic tumor pathway, we divided the differentially expressed genes into S100A16 high-expressed and low-expressed subgroups and conducted a GSEA pathway enrichment analysis. Then, according to its normalized enrichment score, the signaling pathways with the most significant enrichment when S100A16 is up-regulated and down-regulated were selected. GSEA analysis showed that translation elongation, mitochondrial membrane tissue, mitotic division, nucleoside monophosphate biosynthesis, and mismatch repair oxidative phosphorylation were all enriched in the S100A16 high expression phenotype. On the contrary, the regulation of leukocyte system or roll, CAMP-dependent protein kinase activity, camp-mediated negative regulation of signal transduction, glutamate receptor signal transduction pathway, AMPA receptor activity, and excretion showed different enrichments in the S100A16 down-regulation group (Figure 12A). To better understand the role of S100A16 in pancreatic cancer development, we established the protein-protein interaction (PPI) network and performed a statistical analysis and visualization using Cytoscape. The results show that S100A16 interacted mainly with SUCLG1, IDH3A, and SUCLA2 in PC (Figure 12B).

### Correlation Analyses Between S100A16 and Immune Infiltration

**Figure 13:**
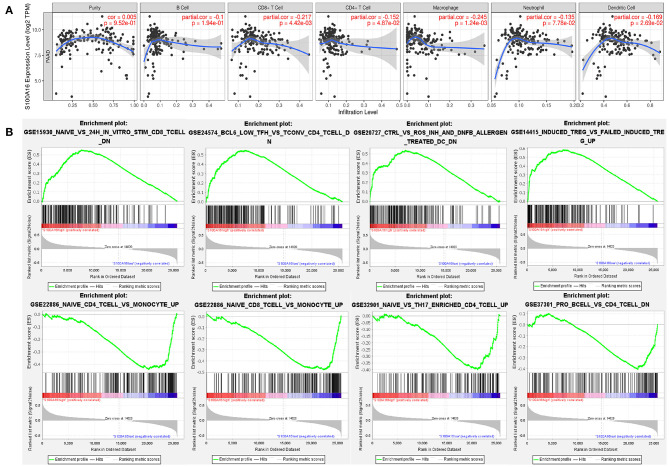
Correlation analysis between the expression level of S100A16 and immune infiltration. **(A)** GSEA analysis showed that naive BCL6, low ctrl induced treg were differentially enriched in the high expression S100A16 phenotype. Naive CD4 T cell, naive CD8 T cell, and naive, pro B cell were differentially enriched in the low expression S100A16 phenotype **(B)**.

**Figure 14:**
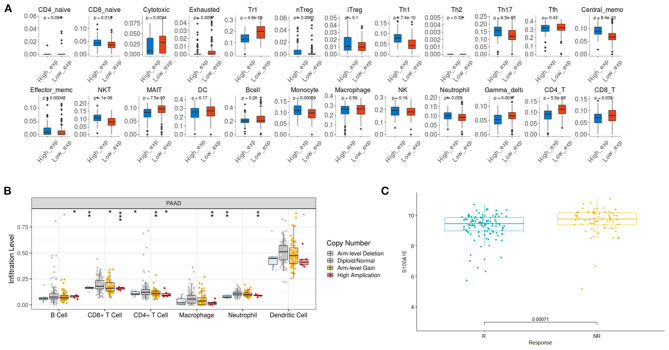
Immune CellAI for S100A16 based on the TCGA database. **(A)** The relationship between the S100A16 copy number variation and infiltration level. *P < 0.05; **P < 0.01; ***P < 0.001 **(B)**. ICB response prediction showed the predicted outcome of immune checkpoint treatment response **(C)**.

According to the TIMER database analysis, CD8+ T cells were negatively correlated with S100A16, suggesting that S100A16 was correlated with tumor immunity (Figure 13A). Univariate COX survival analysis showed that five types of immune cells and S100A16 had significant effects on the survival time of PDAC patients. Multivariate analysis of S100A16 expression and operating system immune osmotic PDAC patients showed age, purity, CD4 T cells, and S100A16 as possible independent prognostic factors for PDAC survival (Supplementary Table 1). With the GSEA analysis of immune-related gene sets, we found S100A16 high expression groups in different data sets of infiltration of immune cells and immune-related cells. In the group with a high expression of S100A16, the differential genes were mainly concentrated in Naive, BCL low TFH, CRTL, and induced Treg, while in the group with a low expression of S100A16, the differential genes were mainly concentrated in Naive CD4 T cells, Naive CD8 T cells, naïve, and Pro B cells (Figure 13B). Immunoassay results on the TCGA database showed that there were significant differences in the expression of S100A16 in naive CD4, CD8, Cytotoxic, exhausted, Tr1, nTreg, Th1, TH17, Central Memo, Effecter Memo, NKT, MAIT, monocyte, gamma, delta, and CD4T (Figure 14A). “ICB response prediction” can be used to output the predicted immune checkpoint response of PDAC patients. This can be used to predict the immune checkpoint response in PDAC patients (Figure 14C). In addition, the change of copy number S100A16 can significantly affect the infiltration level of immune cells in PDAC (Figure 14B), indicating that S100A16 can influence the changes of the immune infiltration level and the number through replication, thus affecting the prognosis of PATIENTS with PDAC. In summary, S100A16 has potential value in pancreatic tumor remission and immunotherapy.

## Discussion

Early stage PDAC has no obvious symptoms and often progresses to advanced PDAC, when detected, with a poor prognosis (Bray et al., 2018). Therefore, finding accurate biomarkers for early diagnosis of PDAC will be helpful for the treatment and prognosis of patients (Leclerc and Vetter, 2015). The S100 protein family may have the potential to be such a biomarker (Bydoun et al., 2018). The S100 family plays a different role in PDAC, S100A11, and S100P are important markers of PDAC, as well as factors contributing to poor prognosis in patients undergoing surgical resection (Ji et al., 2014). S100A2 is also considered a biomarker for poor prognosis of PDAC (Ohuchida et al., 2007). S100A6 in the nucleus could also be an independent prognostic factor for PDAC (Ohuchida et al., 2005). The expression of S100A4 is related to the development and final outcome of the tumor (Che et al., 2015). Studies have shown that S100A16 may be a prognostic factor for colon and lung cancer (Sun et al., 2018; Xu et al., 2019). Our study explored the value of S100A16 in PDAC, and the results showed that S100A16 was more expressed in human pancreatic tissue than in other tissues, with significant differences in PDAC tissue and normal tissues. These results suggest that S100A16 may be differentially expressed in PDAC. GEPIA analyzes the relationship between S100A16 expression and clinical prognosis. Analysis results showed that the prognosis of patients with a high expression of S100A16 and a low expression of S100A16 was significantly different, suggesting that S100A16 can be used as a prognostic molecule of PDAC; multivariate Cox regression analysis showed the same results. These results indicate that S100A16 has the potential to be an important biomarker for the diagnosis and prognosis of PDAC.

S100A16 exerts its biological effects in different tissues and in different ways. For example, S100A16 can exacerbate fibrotic TGF-in tubular epithelial cells by promoting epithelial mesenchymal transformation in renal injury (Sun et al., 2020). S100A16 can also promote the proliferation and invasion of cervical cancer cells. These related biological processes are regulated by the activation of the PI3K/Akt signaling pathway. (Zhang et al., 2020). S100A16 may play a role through tumor suppressor genes p21 and p27 as well as ERK and Akt signaling molecules (Zhu et al., 2016). To date, the mechanism of the S100 protein family in PDAC has not been extensively studied, which may occur through the independent effect of EMT/ZEB1 and IL-6/11-STAT3 signals and aggregation to establish the S100 protein expression pattern, thus promoting the invasion of PDAC (Al-Ismaeel et al., 2019). In our study, KEGG analysis showed that DEGs were concentrated mainly in the pathway related to pancreatic secretory function. Relevant studies have shown that S100A16 can reduce the sensitivity of 3T3-L1 to insulin, overexpression of S100A16 can promote lipid synthesis of 3T3-L1 preadipocytes, inhibiting glucose uptake under insulin stimulation, and cause insulin resistance (Kan et al., 2019). Overexpression of S100A16 in 3T3-L1 cells promotes proliferation and differentiation of 3T3-L1 cells, which is consistent with our findings. These results suggest that S100A16 may play an important role in secretory pancreatic diseases, but whether S100A16 affects the prognosis of PDAC through specific mechanisms remains to be further explored.

In the clinical correlation analysis study, the TCGA database was used to analyze the pathological factors associated with S100A16 in patients with PDAC, and Kaplan-Meier analysis results were used to explain the correlation between S100A16 expression and prognosis in patients with PDAC. Univariate COX analysis was applied to verify the above assumptions and it was found that S100A16 was associated with high risk factors such as age, histological grade type, major treatment response, and radiotherapy. These results suggest that S100A16 can be used as an indicator of disease progression and prognosis in patients with PDAC. However, in the multivariate COX regression analysis of these factors, S100A16 cannot serve as a predicting factor of prognosis (*P* > 0.05). This may be due to the incorporation of the Multivariate COX regression analysis of the weight of the S100A16 dilution. To solve this problem, we used the LASSO regression dimension reduction analysis to filter out the five genes, and built a S100A16-related prognostic model. The risk once again proved that the S100A16 embedded the multivariate COX regression analysis and related genes in their prognostic role. To create a quantitative tool suitable for clinical work to predict OS in patients with PDAC, we also constructed a clinical prediction nomogram model that can more accurately predict the prognosis of patients with PDAC. On the other hand among the genes screened out in the LASSO regression analysis, LncRNA “UCA1” is the oncogene and the UCA1 Prime Function BC through the PI3K/Akt/CREB pathway (Yang et al., 2012). The overexpression of lncRNA UCA1 was associated with drug resistance to chemotherapy drugs such as cisplatin, gemcitabine, 5-Fu, tamoxifen, etc., and after lncRNA-UCA1 was silenced, the drug sensitivity was reversed (Wang et al., 2017). Therefore, whether S100A16 is regulated by LncRNA and plays a role in tumor may be the next potential research direction. However, there were some limitations in our study due to public data sources, as more valuable clinical characteristics could not be further explored. More specifically, the correlation between serological and biochemical parameters, like CA19-9 and serum amylase, and S100A16 in pancreatic cancer, may be one of our next research directions, which requires further data verification through the collection of a large number of clinical samples from PDAC patients.

In this paper, we also explored the correlation between S100A16 expression and immune cell infiltration in PDAC. Relevant studies have shown that many other S100 protein family members are closely related to tumor immune response (Ulas et al., 2017). Once S100 proteins is released into extracellular space, it will be able to interact with multiple immune receptors, like RAGE and Toll-like Receptors (TLRs), and further regulate diverse biological processes including chemokines secretion, tumor cell migration, cell proliferation and tissue repair, etc. (Leclerc et al., 2009; Donato et al., 2013; Gross et al., 2014; Bertheloot and Latz, 2017). S100A8, S100A9, and S100A8/A9 complexes have direct chemotaxis of various immune cells that are involved in keratinocytes inducing pro-inflammatory cytokines and stimulating keratinocytes to produce pro-angiogenic mediators (Halawi et al., 2014). S100A7, S100A12, S100A8, and S100A9 play certain roles in the innate immune response induced by pathogenic bacteria (Kozlyuk et al., 2019). The role of the S100 protein family in tumor immunity is also understood. The expression of S100A9 was related to the expression of CD68 macrophages in a human prostate tumor biopsy. S100A9 in tumors appears to be mainly expressed by CD11b cells (Källberg et al., 2012). In the model of spontaneous breast cancer, blocking the antibody of S100A4 can affect tumor growth and metastasis (Grum-Schwensen et al., 2015). Under abnormal conditions, S100A7, located in the extracellular compartment, can cause the movement of immune cells and tumor cells (Wolf et al., 2008; Kataoka et al., 2012). S100A7 is associated with increased macrophage infiltration during tumor development (Nasser et al., 2012). In addition, S100A8/A9 increases the accumulation of circulating immune cells by enhancing the ability of immune cells to adhere to the endothelium (Wang et al., 2018). These results indicate that the S100 protein family may have an impact on the tumor at different stages of tumor development, which raises some ideas for the further development of tumor immunotherapies. Our results showed that multiple immune cells associated with S100A16 were independent prognostic factors in PDAC patients, and there were significant differences in the immune infiltration of immune cells and immune related cell data sets in the S100A16 high and low expression group. ICB response prediction results showed that the immune checkpoint response was different in patients with PDAC, the change of the S100A16 copy number can significantly affect the level of immune cell infiltration in PDAC. This study found that S100A16, which has not yet been studied, may have a correlation in the immune infiltration mechanism of PDAC, highlighting the potential value of S100A16 in the immunotherapy of PDAC.

In summary, we found increased expression of S100A16 in PDAC. Evidence suggests that it is the independent prognostic factor of pancreatic tumors. The analysis of relevant methods shows that S100A16 is likely to play a certain role in endocrine function of the pancreas. Clinical correlation analysis shows that S100A16 can be used as an indicator value for the diagnosis and prognosis of PDAC patients. We built a comprehensive model to better predict and analyze patient prognosis. Multivariate analysis of S100A16 expression and OS immune infiltration in PDAC patients showed that age, purity, CD4 T cells, and S100A16 may be independent prognostic factors for PDAC. There were differences in the immune checkpoint response in patients with PDAC, and S100A16 has a potential reference value for the remission of PDAC and immunotherapy. This study uses a variety of bioinformatics methods to process and analyze a large number of data, but there are still some limitations. First, all data were from TCGA and ICGC databases, and a small number of cases in the human case group may lead to bias in differential gene results. In future studies, a PDAC database with more data may be a more urgent problem, so the application value of this model needs to be further improved and studied. Moreover, this study lacks further *in vitro* and *in vivo* validation, thus, we will study the function and mechanism of S100A16 through further basic experiments.

## Materials and Methods

### GEPIA Database Analysis

GEPIA database (http://gepia2.cancer-pku.cn/#index) collects gene expression data from TCGA and GTEx, including 9,736 tumor samples and 8,587 normal controls. This database allows for the exploration of gene expression and prognosis in different tumors. We used GEPIA to analyze the expression and prognosis of S100A16 in PDAC in this study.

### Oncomine Database Analysis

Oncomine (www.ONCOMINE.org) is a public database that functions as an analyzer for the expression of various tumor genes in different transcriptional profiling datasets. S100A16 were input into the database to detect its expression level in various tumors including pancreatic adenocarcinoma. Analysis condition setting: Gene name: S100A16. Analysis Type: Cancer vs. Normal and Cancer vs. Cancer. *P*-value < 0.05 and Fold Change ≥ 1.5 was considered significant.

### Screening of DEGs

The RNA-seq data of TCGA_PAAD (source: https://portal.gdc.cancer.gov/; *n* = 182) and ICGC_AU_PAAD (source: https://daco.icgc.org/; *n* = 91) cohorts were applied to find DEGs between S100A16 high and low expression subgroups, which defined by the median expression level of S100A16. DEGS were screened using R package “DESeq2” and heatmaps were plotted using R package “heatmap” in two cohorts, respectively. *P* < 0.05, log FC > 1.5 was defined as significantly upregulated DEGS, while *P* < 0.05, log FC < −1.5 was defined as significantly downregulated DEGS. Then, the intersection of the DEGs obtained by TCGA and ICGC cohort was defined as the final DEGs in our study.

### GO and KEGG Pathway Analysis

GO analysis is divided into three parts, including cellular component, molecular Function, and biological Process, explaining the biological function of certain genes from different aspects. KEGG is an analysis method used to discover which biological pathways certain genes are enriched in. Using _ to perform GO and KEGG pathway analysis based on DEGS with low expression vs. high expression of S100A16.

### Construction of “S100A16 Risk Score” Predicting System

TCGA_PAAD cohort with complete clinical information was applied in this part. Correlation between multiple clinical characteristics and the expression level of S100A16 was analyzed by boxplot. Univariate and multivariate Cox regression analysis was then adopted to preliminarily detect the relationship between patient survival and clinical characteristics along with the expression of S100A16. Then, to further delineate the impact of S100A16 and its related genes on the prognosis of patients, we applied the following protocol: First, Pearson correlation analysis and univariate cox analysis was implemented to screen S100A16-related prognostic genes. Then, least absolute shrinkage and selection operator (LASSO) Cox regression, achieved by R package “glmnet” was conducted to perform dimension reduction. After these procedures, a S100A16-related gene set composed of five genes was successfully developed. The five genes were “SERPINB1”,“RAB27B”, “MGLL”, “ANKRD22”, and “UCA1”. Along with S100A16, these 6 genes were included to calculate a **S100A16** risk score for each patient using the following formula:

(1) $$\begin{array}{r} Risk\;Score=\;\overset{n}{\underset{i=1}{\sum }}Coef_{i}\;*\;x_{i} \end{array}$$

where Coefi means the coefficients for each gene; xi is the FPKM value of each gene.

Risk scores were computed for all patients included in our study. To further obtain a clinical model with predicting values, another multivariate Cox regression was applied to establish a nomogram, integrating all clinical characteristics that had HR >1 (or < −1) and *P* < 0.05. The calibration plots show the prognostic predictive accuracy of the nomogram.

### Immunohistochemical Staining (IHC) for S100A16

IHC staining of S100A16 was conducted on paired tumors and adjacent normal tissues for three PDAC cases. IHC staining was carried out using the Histostain-Plus kit according to the manufacturer's protocol. Briefly, antigen retrieval was conducted by heating the sections in boiling sodium citrate buffer for 20 min. After 3% hydrogen peroxide and BSA blocking, the tissues were incubated with 1:200 diluted S100A16 antibody (©Affinity Biosciences LTD, OH, USA) at 4°C overnight. After washing, the tissues were incubated with 1:200 diluted HRP-conjugated secondary antibody (Santa Cruz Biotechnology, Santa Cruz, CA). IHC signal was developed by DAB substrate, and counter-stained by hematoxylin. Random fields at 20× and 40× magnification were captured per sections for evaluation.

### GSEA Analysis

Gene set enrichment analysis (GSEA) provided by the JAVA program (Version 4.0.3) with MSigDB v6.1 was applied to explore the downstream biological processes affected by differential expression of S100A16. Patients in the TCGA_PAAD cohort were divided into two groups by the standard of “Screening DEGs”; 25,880 genes were enrolled into the GSEA process. Hallmark gene set “c5.bp.v7.0.symbols.gmt” was used in this study. Gene sets which obtained the highest Enrichment Score (ES) with a normalized *p*-value < 0.05, and a false discovery rate (FDR) of < 0.25, were considered significantly enriched.

### Construction of Protein-Protein Interaction (PPI) Network

Level-1 and level-2 PPI analysis for S100A16 was performed using STRING database (https://www.string-db.org/). This database provided us with information on the interaction score between proteins. After downloading the results in TSV format, a PPI network was established by Cytoscape software (NIH, National Resource for Network Biology) to further cluster proteins into different modules and to achieve visualization.

### Immune Infiltration Analysis

Correlation between S100A16 and immune infiltration were analyzed by three different tools based on the TCGA_PAAD cohort, which were TIMER (https://cistrome.shinyapps.io/timer/), ImmuneCellAI (http://bioinfo.life.hust.edu.cn/web/ImmuCellAI/), and GSEA (Hallmark gene set “c5. immunologic signature gene sets.gmt”). The significance of immune cells in the prognosis of PDAC patients were analyzed by TIMER. Moreover, TIMER provided an analysis of clinical correlation between immune cell infiltration and patient survival. ImmuneCellAI could predict a patient's response to immune checkpoint blocker (ICB) treatment based on transcriptomic profiling.

## Data Availability Statement

Publicly available datasets were analyzed in this study. This data can be found here: b1 TCGAdownload by R package “TCGAbiolinks,” data source: GDC data portal (https://portal.gdc.cancer.gov/projects/TCGA-PAAD) 2 ICGC: https://dcc.icgc.org/releases/current/Projects/PACA-AU/b.

## Author Contributions

HZ and XY: conceptualization and funding acquisition. WG and GT: methodology, validation, and formal analysis. WG: software and writing—original draft preparation. GT: data curation and writing—review and editing. YL, YD, BX, and LN: project administration. All authors have read and agreed to the published version of the manuscript.

## Conflict of Interest

The authors declare that the research was conducted in the absence of any commercial or financial relationships that could be construed as a potential conflict of interest.
