# Supplementary material for: Expressional and Prognostic Value of S100A16 in Pancreatic Cancer Via Integrated Bioinformatics Analyses
Source: Front Cell Dev Biol. 2021 Apr 12;9:645641. doi: 10.3389/fcell.2021.645641 (PMC8072221; doi:10.3389/fcell.2021.645641)
Supplement: Supplementary file 1 [file Data_Sheet_1.zip › Table 1.DOCX]

**Supplementary Table 1.** Multivariate analyses of the correlation of S100A16 expression and immune infiltrates with OS among PDAC patients.

|  |  |  |  |  |  |
| --- | --- | --- | --- | --- | --- |
|  | coef | HR | 95%CI_u p.value | sig |  |
| Age | 0.023 | 1.023 | 1.000 1.047000e+00 | 0.052 | · |
| gendermale | -0.263 | 0.768 | 0.494 1.195000e+00 | 0.243 |  |
| raceBlack | -0.444 | 0.641 | 0.131 3.139000e+00 | 0.584 |  |
| raceWhite | 0.445 | 1.56 | 0.593 4.104000e+00 | 0.367 |  |
| stage2 | 0.019 | 1.02 | 0.425 2.448000e+00 | 0.965 |  |
| stage3 | -1.052 | 0.349 | 0.041 2.969000e+00 | 0.335 |  |
| stage4 | -0.841 | 0.431 | 0.082 2.267000e+00 | 0.321 |  |
| Purity | -0.982 | 0.375 | 0.138 1.014000e+00 | 0.053 | · |
| B_cell | 4.604 | 99.892 | 0.249 4.008095e+04 | 0.132 |  |
| CD8_Tcell | 3.097 | 22.124 | 0.026 1.914803e+04 | 0.37 |  |
| CD4_Tcell | -7.545 | 0.001 | 0.000 1.000000e+00 | 0.05 | · |
| Macrophage | -2.406 | 0.09 | 0.000 9.650000e+01 | 0.499 |  |
| Neutrophil | 10.315 | 30183.754 | 0.013 7.199803e+10 | 0.169 |  |
| Dendritic | -2.885 | 0.056 | 0.001 2.188000e+00 | 0.123 |  |
| S100A16 | 0.431 | 1.538 | 1.146 2.065000e+00 | 0.004 | ** |
| Rsquare= 0.205 (max possible= 9.91e-01 ) | | | | |  |
| Likelihood ratio test p= 9.21e-04 | | | | |  |
| Wald test p= 1.99e-02 | | | | |  |
| Score (logrank) test p= 1.04e-02 | | | | |  |
