# Supplementary figures and images for: Expressional and Prognostic Value of S100A16 in Pancreatic Cancer Via Integrated Bioinformatics Analyses
Source: Front Cell Dev Biol. 2021 Apr 12;9:645641. doi: 10.3389/fcell.2021.645641 (PMC8072221; doi:10.3389/fcell.2021.645641)

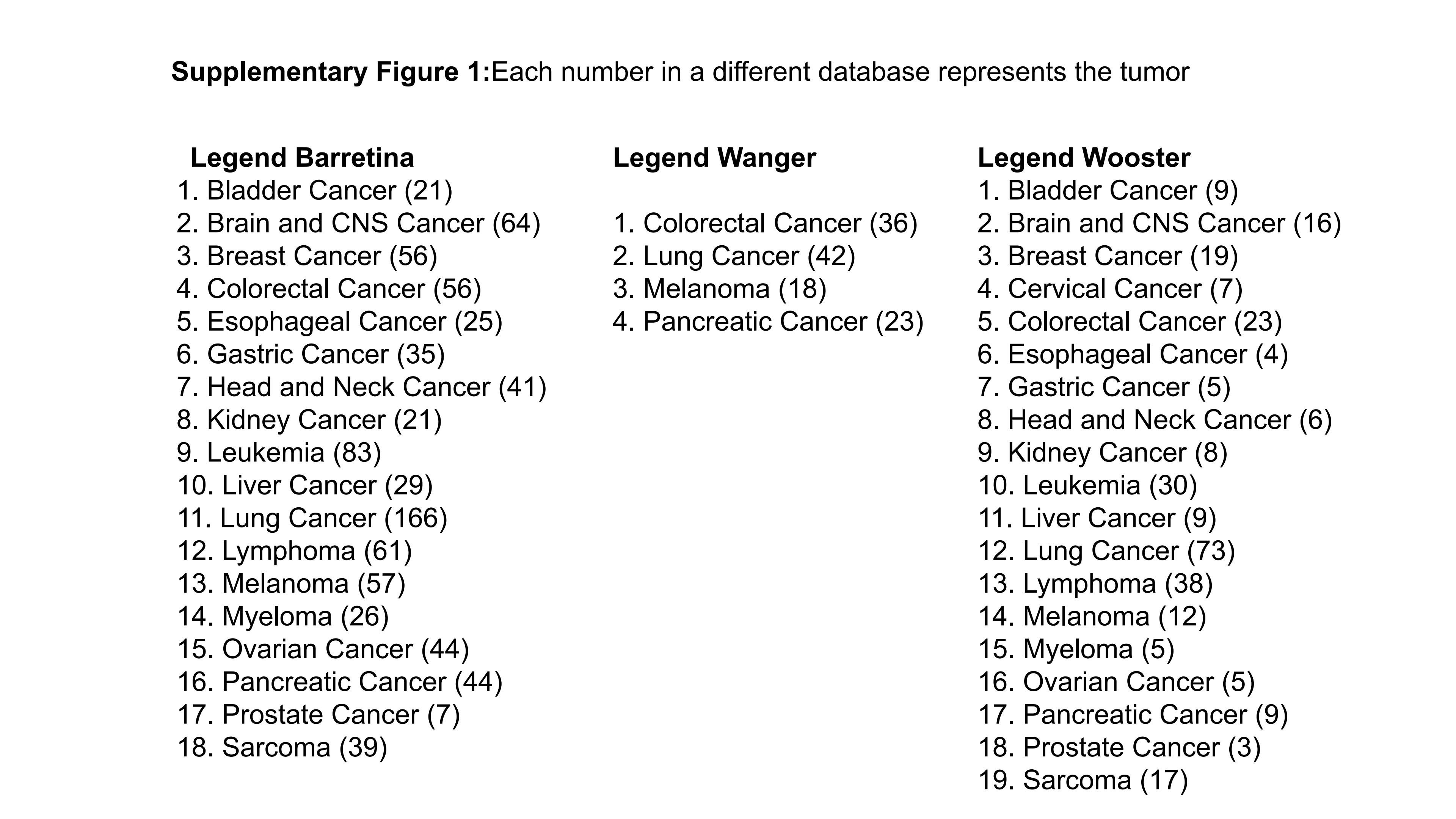

Supplement: Supplementary file 1 [file Data_Sheet_1.zip › Image 1.JPEG]

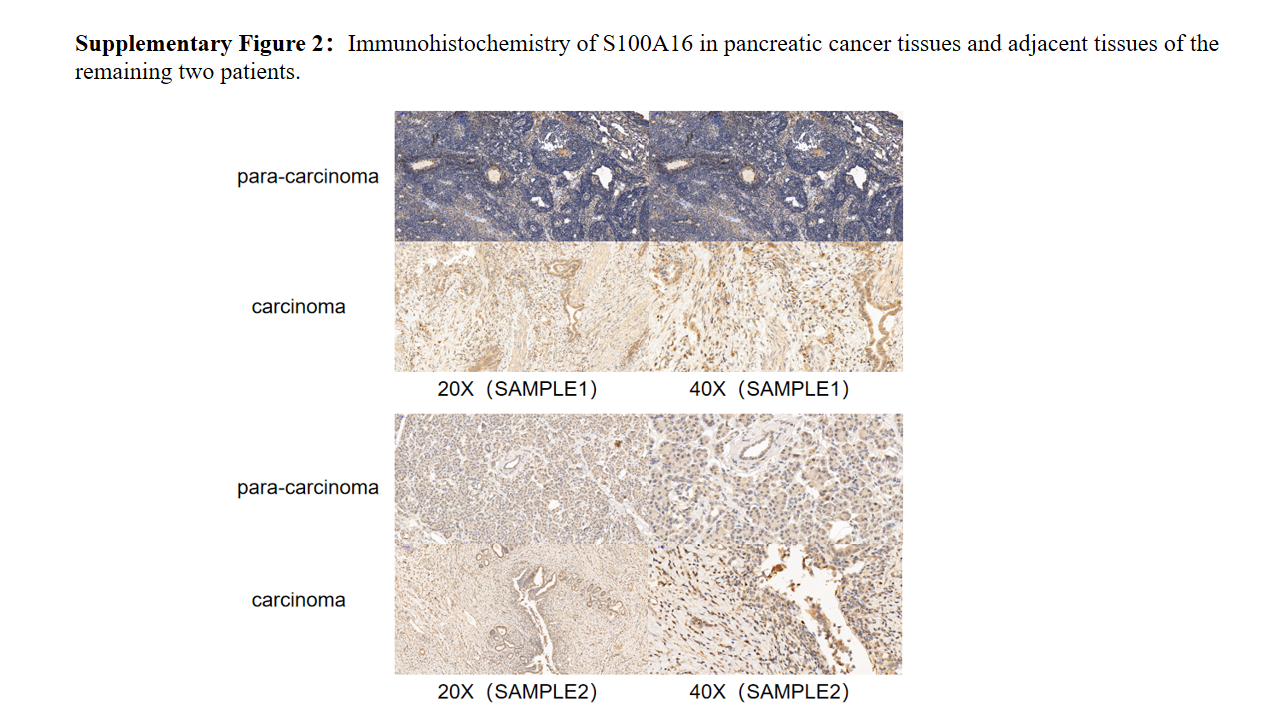

Supplement: Supplementary file 1 [file Data_Sheet_1.zip › Image 2.TIF]
